# Postoperative brachial artery entrapment associated with pediatric supracondylar fracture of the humerus: a case report

**DOI:** 10.1186/s13256-017-1240-4

**Published:** 2017-03-14

**Authors:** David Latz, Jan Grassmann, Erik Schiffner, Sebastian Gehrmann, Mansur Duran, Joachim Windolf, Pascal Jungbluth

**Affiliations:** 1 Department of Trauma and Hand Surgery, University Hospital, Moorenstrasse 5, 40225 Düsseldorf, Germany; 2 Department of Vascular Surgery and Kidney Transplantation, University Hospital, Moorenstrasse 5, 40225 Düsseldorf, Germany

**Keywords:** Case report, Pediatric supracondylar fracture of the humerus, Gartland type III, Neurovascular complications, Entrapment of the brachial artery, Open K-wire fixation

## Abstract

**Background:**

Severely displaced supracondylar fractures of the humerus in children are frequently associated with complications including neurovascular injuries, non-union, or compartment syndrome. In the current literature, no report exists about postoperative brachial artery entrapment in combination with an inconspicuous preoperative neurovascular examination.

**Case presentation:**

We present a case of a 6-year-old white boy with a pulseless radial and ulnar artery after open reduction and internal fixation of a severely displaced supracondylar fracture of his right humerus (Gartland type III) using four K-wires. Remarkably, the preoperative neurovascular examination was inconspicuous. Doppler ultrasound of his brachial artery revealed no pulse when his elbow was in flexion and a faint pulse when it was in full extension 10 hours postoperatively. Revision surgery was performed immediately. On intraoperative examination, a kinking of his brachial artery caused by an entrapment of the tunica externa in the reduced fracture was seen and the artery was released by microsurgical arteriolysis immediately. At the final follow-up examination, positive palpable pulse with good capillary filling and, according to Flynn’s criteria, an excellent recovery of elbow function was observed 3 months postoperatively.

**Conclusions:**

This case demonstrates a rare complication of postoperative artery entrapment with inconspicuous preoperative neurovascular examination. It strongly emphasizes the need for a standardized postoperative neurovascular assessment with fully flexed as well as fully extended elbow.

## Background

Supracondylar fractures of the humerus are common elbow fractures in children [1–4]. Gartland’s classification based on the degree of displacement of the distal fragment is used frequently [5]. Fractures with complete displacement of the distal fragment (Gartland type III) are frequently associated with various complications such as neurovascular injuries, non-union, or compartment syndrome that can lead to a Volkmann’s contracture or even amputation of the affected limb [3, 5–8]. Preoperative neurovascular assessment scores (for example, Liverpool Upper-limb Fracture Assessment) have already been proposed to optimize evaluation of children presenting with upper limb injuries in Accident and emergency (A&E) departments [9]. The following case presents a rare vascular complication despite an inconspicuous preoperative neurovascular examination and emphasizes the importance of a thorough postoperative neurovascular assessment.

## Case presentation

**Fig. 1:**
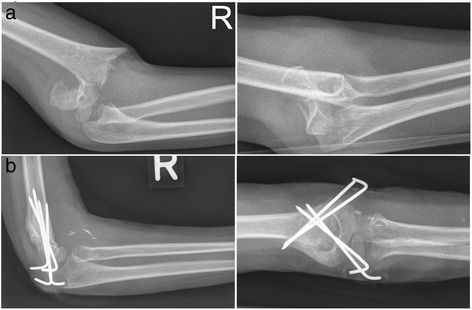
Radiographs in two planes: **a** preoperative and **b** postoperative after 6 weeks

A 6-year-old white boy presented to our A&E department after a fall on his extended right arm from a height of 1 m. An initial examination revealed a severely displaced right elbow without penetration of the skin. A physical examination was hindered by his anxiety and pain. However, according to Liverpool Upper-limb Fracture Assessment, his neurovascular examination was reported as unremarkable. Palpable radial and ulnar pulses as well as no sensorimotor deficit were obtained. Radiographs in two planes showed a posterolaterally displaced supracondylar fracture of his humerus classified as Gartland type III (Fig. 1a). Surgical efforts under general anesthesia were initiated immediately. An initial attempt of closed reduction was unsuccessful. Hence, open reduction and internal fixation using four K-wires were performed immediately (Fig. 1b). No intraoperative complications were reported. On postoperative examination, arterial pulses were palpable in elbow extension with unaffected capillary filling. His arm was immobilized in a long-arm cast with elbow flexion of 90°. Reevaluation of his arterial pulses after immobilization was not documented.

**Fig. 2:**
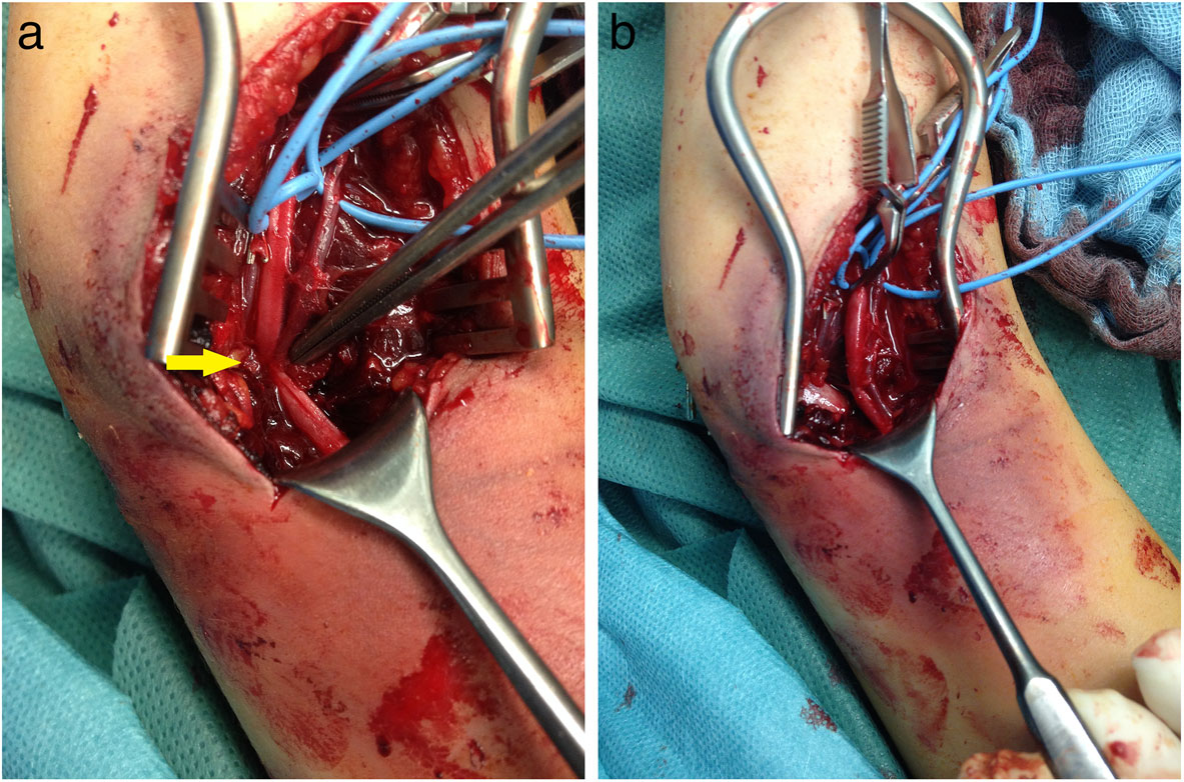
**a** Entrapment of the brachial artery (*yellow arrow*) and **b** after microsurgical arteriolysis

Ten hours after surgery, he complained of acute pain in his right forearm. A physical examination revealed a coldness of his right forearm with poor capillary filling and absent arterial pulses while immobilized in the long-arm cast with elbow flexed to 90°. The cast was removed and Doppler ultrasound revealed that there was no pulse while his elbow was in flexion and faint pulses with his elbow in extension. The immediately performed revision surgery (transverse arteriotomy and exploration with a Fogarty catheter) revealed no arterial thrombosis. After extending the surgical approach a kinking of his brachial artery caused by an entrapment of the tunica externa in the reduced fracture was observed (Fig. 2a). It was released with microsurgical arteriolysis (Fig. 2b). Immediately, his radial and ulnar pulses were palpable with good capillary filling.

**Fig. 3:**
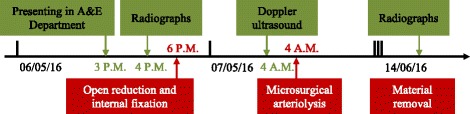
Timeline. *A&E* Accident and emergency

Postoperative immobilization was achieved using a long-arm cast with the elbow at 90° flexion for 6 weeks. Implant removal at 6 weeks was followed by intensive physical therapy to increase the range of motion (ROM). At the final follow-up examination at 3 months, the subjective elbow function had fully recovered. He had positive palpable pulses, a warm forearm with good capillary filling and, according to Flynn’s criteria [2, 10], an excellent recovery of elbow function as compared to the healthy contralateral side with 100 % ROM in extension/flexion 10-0-150°, pronation/supination 90-0-90°, and no loss of carrying angle were observed. Figure 3 gives an overview of the case.

## Discussion

Gartland type III fractures are highly correlated with primary neurovascular lesions [11–14]. Posteromedial displacement is associated with radial nerve injuries, whereas posterolateral displacement is associated with median nerve injuries. Brachial artery injuries occur in both types of displacement equally [15, 16]. Typical vascular injuries are intimal tear, thrombus formation, and division or spasm of the vessel [17, 18]. The initial therapy of pediatric supracondylar fractures with an absent radial pulse and a cold white hand is closed reduction and fixation with K-wires. If the initial attempt is unsuccessful in restoring the pulse, open reduction and vascular exploration is mandated [19]. Management of children with absence of their radial pulse with a well-perfused hand is still controversial [19–21]. Remarkably, no difference between early surgical treatment (8 hours or less following injury) and delayed surgical treatment (more than 8 hours following injury), with regard to the perioperative complication rates, is reported [22]. Due to serious primary neurovascular lesions and difficult physical examination in young patients, a standardized neurovascular assessment has already been proposed [9]. In the current literature, only a few reports about delayed neurovascular complications exist [23–25]. In fact, an excellent clinical long-term outcome can be expected if postoperative circulatory failure is treated immediately [24]. In general, artery entrapment syndromes, for example caused by a humeral bony spur, are usually clinically unapparent and symptoms only occur with specific movements, such as extension or flexion of the elbow [26, 27]. Arguably, a thorough postoperative neurovascular assessment with fully flexed as well as fully extended elbow can detect a postoperative artery entrapment.

## Conclusions

This case demonstrates a rare complication of a postoperative brachial artery entrapment despite inconspicuous preoperative neurovascular examination. This strongly emphasizes the need for a standardized postoperative neurovascular assessment in a full range of elbow motion.

## References

[1] Davis RT, Gorczyca JT, Pugh K. Supracondylar humerus fractures in children. Comparison of operative treatment methods. Clin Orthop Relat Res. 2000;376:49–55. 10906857

[2] Sahu RL. Percutaneous K-wire fixation in paediatric supracondylar fractures of humerus: A retrospective study. Niger Med J. 2013;54:329–34. doi:10.4103/0300-1652.122355. PMC3883234 24403712

[3] Houshian S, Mehdi B, Larsen SM. The epidemiology of elbow fracture in children: analysis of 355 fractures, with special reference to supracondylar humerus fractures. J Orthop Sci. 2001;6:312–5. doi:10.1007/s007760100024. 11479758

[4] James D, Gajendran M, Paraseth TK. Functional and radiological correlation in surgically managed severe supracondylar humerus fracture in a pediatric cohort using pediatric outcomes data collection instrument upper extremity scale: A report from a level V trauma center in rural Central India. CHRISMED J Health Res. 2017;4:43.

[5] Alton TB, Werner SE, Gee AO. Classifications in brief: the Gartland classification of supracondylar humerus fractures. Clin Orthop Relat Res. 2015;473:738–41. doi:10.1007/s11999-014-4033-8. PMC4294919 25361847

[6] von Laer LR. Der radiale Fixateur externe zur Behandlung suprakondylarer Humerusfrakturen im Wachstumsalter. Oper Orthop Traumatol. 1997;9:265–76. doi:10.1007/s00064-006-0098-1. 17004137

[7] Dormans JP, Squillante R, Sharf H. Acute neurovascular complications with supracondylar humerus fractures in children. J Hand Surg [Am]. 1995;20:1–4. doi:10.1016/S0363-5023(05)80046-2. 7722246

[8] Gosens T, Bongers KJ. Neurovascular complications and functional outcome in displaced supracondylar fractures of the humerus in children. Injury. 2003;34:267–73. 10.1016/s0020-1383(02)00312-1 12667778

[9] Mayne AI, Perry DC, Stables G, *et al.* Documentation of neurovascular status in supracondylar fractures and the development of an assessment proforma. Emerg Med J. 2013;30:480–2. doi:10.1136/emermed-2012-201293. 22761511

[10] Flynn JC, Matthews JG, Benoit RL. Blind pinning of displaced supracondylar fractures of the humerus in children. Sixteen years’ experience with long-term follow-up. J Bone Joint Surg Am. 1974;56:263–72. 4375679

[11] Shaw BA, Kasser JR, Emans JB, *et al.* Management of vascular injuries in displaced supracondylar humerus fractures without arteriography. J Orthop Trauma. 1990;4:25–9. 10.1097/00005131-199003000-00004 2313426

[12] Abbott MD, Buchler L, Loder RT, *et al.* Gartland type III supracondylar humerus fractures: outcome and complications as related to operative timing and pin configuration. J Child Orthop. 2014;8:473–7. doi:10.1007/s11832-014-0624-x. PMC4252275 25381182

[13] Rasool MN, Naidoo KS. Supracondylar fractures: posterolateral type with brachialis muscle penetration and neurovascular injury. J Pediatr Orthop. 1999;19:518–22. 10.1097/00004694-199907000-00019 10413005

[14] Mooney III JF, Hosseinzadeh P, Oetgen M, *et al.* AAOS Appropriate Use Criteria: Management of Pediatric Supracondylar Humerus Fractures With Vascular Injury. J Am Acad Orthop Surg. 2016;24:e24–8. 10.5435/JAAOS-D-15-00702 26803547

[15] Eren A, Güven M, Erol B, *et al.* Correlation between posteromedial or posterolateral displacement and cubitus varus deformity in supracondylar humerus fractures in children. J Child Orthop. 2008;2:85–9. doi:10.1007/s11832-008-0088-y. PMC2656790 19308586

[16] Campbell CC, Waters PM, Emans JB, *et al.* Neurovascular injury and displacement in type III supracondylar humerus fractures. J Pediatr Orthop. 1995;15:47–52. 10.1097/01241398-199501000-00011 7883927

[17] Mohammadzadeh MA, Mohammadzadeh M, Mohammadzadeh A, *et al.* Arterial damage accompanying supracondylar fractures of the humerus. Trauma Mon. 2012;16:160–3. doi:10.5812/kowsar.22517464.3273. PMC3989565 24749093

[18] Kumar R, Trikha V, Malhotra R. A study of vascular injuries in pediatric supracondylar humeral fractures. J Orthop Surg (Hong Kong). 2001;9:37–40. 10.1177/230949900100900208 12118129

[19] Garbuz DS, Leitch K, Wright JG. The treatment of supracondylar fractures in children with an absent radial pulse. J Pediatr Orthop. 1996;16:594–6. 10.1097/00004694-199609000-00009 8865043

[20] Korompilias AV, Lykissas MG, Mitsionis GI, *et al.* Treatment of pink pulseless hand following supracondylar fractures of the humerus in children. Int Orthop. 2009;33:237–41. doi:10.1007/s00264-007-0509-4. PMC2899233 18365192

[21] Badkoobehi H, Choi PD, Bae DS, *et al.* Management of the pulseless pediatric supracondylar humeral fracture. J Bone Joint Surg Am. 2015;97:937–43. 10.2106/JBJS.N.00983 26041856

[22] Mehlman CT, Strub WM, Roy DR, *et al.* The effect of surgical timing on the perioperative complications of treatment of supracondylar humeral fractures in children. J Bone Joint Surg Am. 2001;83-A:323–7. 10.2106/00004623-200103000-00002 11263634

[23] Ege T, Turkkan S, Gunay C, *et al.* Late onset brachial artery thrombosis and total temporary peripheral neuropathy in a child with humerus supracondylar fracture: a case report. Ulus Travma Acil Cerrahi Derg. 2015;21:75–8. doi:10.5505/tjtes.2015.92072. 25779718

[24] Reigstad O, Thorkildsen R, Grimsgaard C, *et al.* Supracondylar fractures with circulatory failure after reduction, pinning, and entrapment of the brachial artery: excellent results more than 1 year after open exploration and revascularization. J Orthop Trauma. 2011;25:26–30. doi:10.1097/BOT.0b013e3181db276a. 21085027

[25] Pandey K, Maravi L, Turkar R. Management of postoperative vascular compromise in supracondylar fracture of the humerus in children. Orthop J MP Chapter. 2015;21(2):70.

[26] Meda N, Verma H, Tripathi RK. Ischemic brachial artery entrapment syndrome by supracondylar humeral bony spur. J Vasc Surg Cases. 2015;1:116–9. 10.1016/j.jvsc.2015.03.009 PMC6849887 31724601

[27] Halha H, Enon B, Chevalier J-M, *et al.* Brachial artery entrapment: Compression by the supracondylar process. Ann Vasc Surg. 1987;1:479–82. doi:10.1007/bf02732675. 3504360

